# Cardiac injuries in blunt chest trauma

**DOI:** 10.1186/1532-429X-11-35

**Published:** 2009-09-17

**Authors:** Marina Huguet, Catalina Tobon-Gomez, Bart H Bijnens, Alejandro F Frangi, Marius Petit

**Affiliations:** 1 Department of Magnetic Resonance Imaging of Cetir Sant Jordi, Barcelona, Spain; 2 Networking Research Center on Bioengineering, Biomaterials and Nanomedicine, Barcelona, Spain; 3 Center for Computational Imaging and Simulation Technologies in Biomedicine, Universitat Pompeu Fabra, Barcelona, Spain; 4 Institució Catalana de Recerca i Estudis Avançats, Barcelona, Spain; 5 Centro Cardiovascular Sant Jordi, Barcelona, Spain

## Abstract

Blunt chest traumas are a clinical challenge, both for diagnosis and treatment. The use of Cardiovascular Magnetic Resonance can play a major role in this setting. We present two cases: a 12-year-old boy and 45-year-old man. Late gadolinium enhancement imaging enabled visualization of myocardial damage resulting from the trauma.

## Background

Blunt chest traumas are a clinical challenge, both for diagnosis and treatment, since they are often associated with substantial cardiac injury [1]. If not recognized and treated promptly, it may have severe, or even fatal, complications for the patient due to myocardial herniation [2]. Myocardial contusion usually shows enzyme rises, electrocardiographic abnormalities and contractile dysfunction [1]. Since these symptoms can be similar for acute peri-traumatic myocardial infarction, a correct diagnosis may be difficult. The use of Cardiovascular Magnetic Resonance (CMR) can play a major role in diagnosing the etiology of the cardiac abnormalities in this setting.

## Case Presentation

### Patient Description

We present two cases where the use of CMR is illustrated for the diagnosis and understanding of cardiac injury. The first patient was a 12-year-old boy referred to our center after a blunt thoracic-abdominal trauma by a rollover vehicle accident at the age of six. Although he was initially asymptomatic, a subsequent tachycardia was noted. An echocardiogram, acquired two years after the trauma, revealed a left midventricular aneurysm with loss of myocardium affecting the septal and posteriorlateral walls.

The second patient was a 45-year-old man who suffered from a blunt chest trauma after precipitating into a trench in which he was buried by construction material and lost consciousness. His echocardiogram showed a ventricular-septal defect with a left to right shunt. The CMR was performed the day after the trauma.

### Examination

Both patients underwent an CMR examination to estimate the severity of myocardial damage using a 1.5 T scanner (Signa CVi-HDx, GE Medical Systems, Waukesha, WS) with a dedicated cardiac coil. The protocol included balanced steady-state free precession gradient-echo images (CINE) and late gadolinium enhancement (LGE) inversion recovery images (after IV administration of 0.2 mmol/kg of gadopentate dimeglumine contrast).

**Figure 1:**
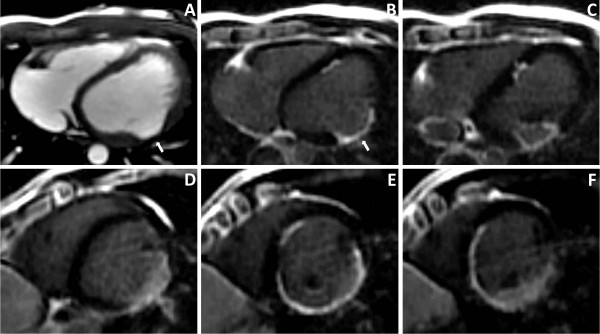
**Patient 1**. A: CINE axial image showing the mid-ventricular posterolateral aneurysm (arrows). B-F: LGE images (B-C: axial; D-F short-axis) showing a helical pattern of enhancement (most probably fibrosis), going from the insertion of the right-ventricular moderator band up to the insertion of the papillary muscle in the left ventricle.

**Figure 2:**
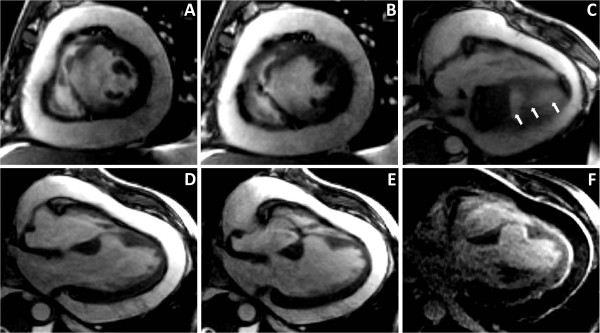
**Patient 2**. CINE images in short-axis at end diastole (A) and end systole (B) and in four-chamber view (C-E) showing the septal rupture at the moderator band insertion and the induced left-to-right shunt and secondary haemopericardium. The inferior wall damage can be depicted in C (arrows). F: the LGE image showing no obvious contrast enhancement in the acute phase.

**Figure 3:**
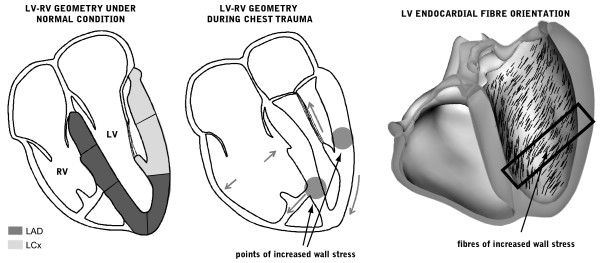
**Schematic representation of the mechanism inducing myocardial damage in a blunt chest trauma**. Left: the ventricular geometry under normal conditions. The shades indicate the coronary territories. The middle panel shows the change in geometry during chest trauma. The arrows represent the direction of the large forces induced by the sudden increase in intrathoracic pressure. The circles indicate the areas of high mechanical stress, where rupture is most likely. The right panel shows the endocardial (oblique) muscle fibre orientation, clearly indicating the path connecting the moderated band insertion with the papillary muscle heads where a chest trauma induces increased wall stress.

The main structural abnormality observed in both patients was the loss of myocardium. The first patient (Figure 1) developed a posterolateral mid-wall aneurism without wall rupture (Figure 1). The LGE images revealed a helical pattern of enhancement, starting at the lower midseptum, along the inferior wall up to the posterolateral wall above the insertion of the papillary muscle (Figure 1B-F). This contrast distribution does not correspond to any particular coronary territory. The second patient (Figure 2) had a rupture of the interventricular septum and the inferior wall with a secondary haemopericardium. The location of septal thinning and the inferior wall rupture are possible points of increased wall stress, as depicted in Figure 3. The LGE images showed no marked contrast enhancement (Figure 2F).

## Discussion

The patterns of myocardial damage visible from these images are consistent with previously reported injury sites [3,4]. In Figure 3, a possible mechanism is presented for explaining the observed rupture. We hypothesize that the sudden increase in intrathoracic pressure during trauma [5] causes the right ventricular pressure to rise and the cavity to expand. This fastly displaces the right ventricular free wall outward, stretching the moderator band and generating a point of high wall stress around its septal insertion. Additionally, the induced increase in left ventricular pressure closes the mitral valve and stretches the tendinous chords and papillary muscles, creating a higher wall stress around the insertion of the papillary muscles. The increased stress from the right ventricular moderator band and the chordal apparatus puts the connecting (helical) endocardial muscle fibres, which run along the septal, inferior and lateral wall, from apex towards the base (Figure 3 right), under extreme wall stress, resulting in fibre damage, as observed in patient 1.

Finally, the lack of LGE in the second patient might be due to either: the acute state of the injury or the strong loss of myocardium resulting in no-reflow [6]. However, further investigation is required to establish the prevalence of this condition among patients.

CMR has the potential to distinguish acute infarction from myocardial contusion, since it allows for a three-dimensional evaluation of the myocardial injury caused by blunt chest trauma. The use of CINE images can depict motion abnormalities and myocardial rupture, while the pattern observed in LGE images can describe the extent of injury (helical pattern vs. coronary territory).

## Conclusion

In summary, CMR imaging enables to visualize the typical pattern of myocardial damage resulting from a blunt chest trauma, thus enabling to make an accurate evaluation of the induced injuries. It can be used to differentiate myocardial contusion from a peri-traumatic myocardial infarction.

## Consent

Written informed consent was obtained from the patients for publication of this case report and any accompanying images.

## Competing interests

The authors declare that they have no competing interests.

## Authors' contributions

MH and CTG contributed equally to this paper and should be considered joint first authors. They drafted the manuscript, and interpreted CMR images. MH was responsible for CMR acquisition. BHB was responsible for the idea for the manuscript and wrote the mechanism interpretation of the manuscript. AFF provided access to the computational atlas for fiber orientation interpretation. MP helped write and rewrote the manuscript. All authors read and approved the final manuscript.
